# Acute myocardial infarction in a young woman with ulcerative colitis: A case report and literature review

**DOI:** 10.1097/MD.0000000000008885

**Published:** 2017-11-27

**Authors:** Yong Zhang, Xuezeng Hao, Xiangying Zheng, Huaibing Zhao, Wei Zhang, Lijing Zhang

**Affiliations:** a Department of Cardiology, Dongzhimen Hospital, The First Affiliated Hospital of Beijing University of Chinese Medicine; b Department of Rehabilitation and Stroke Center, Dongzhimen Hospital, The First Affiliated Hospital of Beijing University of Chinese Medicine, Beijing, China.

**Keywords:** acute myocardial infarction, ulcerative colitis, young woman

## Abstract

**Rationale:**

Myocardial infarction due to nonatherosclerotic coronary thrombosis in young woman with ulcerative colitis is rare.

**Patient concerns:**

A 23-year-old Chinese woman with a 3-year history of ulcerative colitis was admitted to the coronary care unit due to prolonged chest pain.

**Diagnoses:**

Myocardial infarction due to nonatherosclerotic coronary thrombosis was diagnosed in this young woman.

**Lessons:**

Coronary artery thrombosis in ulcerative colitis is a serious condition and can occur in the young population.

## 1 Introduction

The authors report a rare case of myocardial infarction (MI) due to nonatherosclerotic coronary thrombosis in a young woman with ulcerative colitis (UC).

## 2 Case report

**Figure 1:**
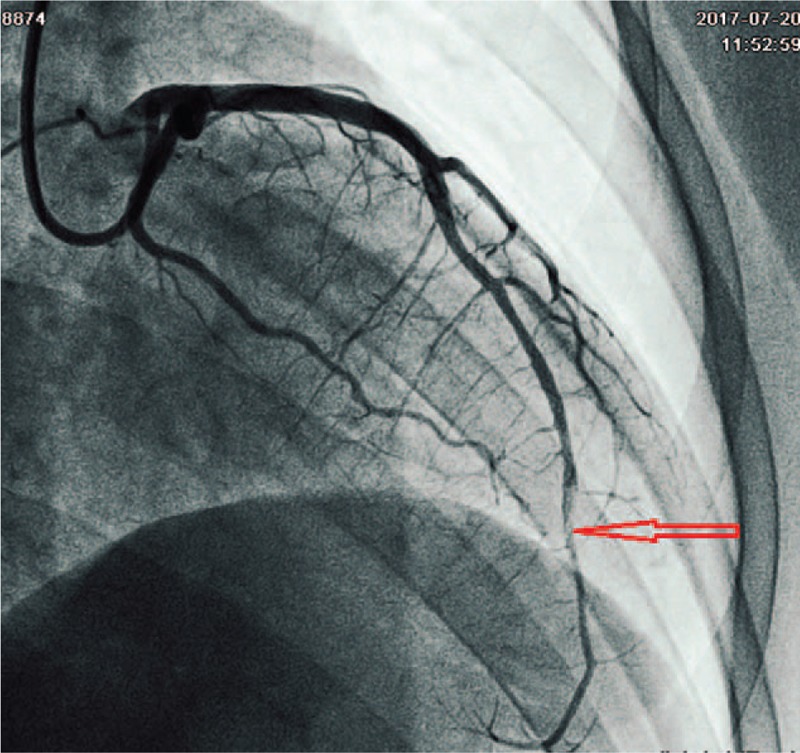
Echocardiographic examination revealed a moderate echogenic mass at the apex.

**Figure 2:**
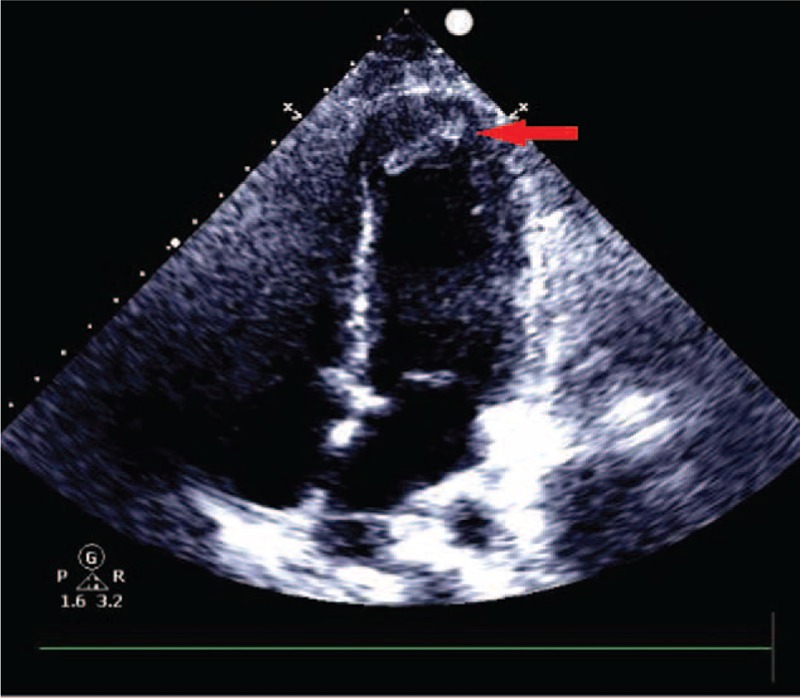
Coronary angiography showed smooth angiographic appearance and a well-formed thrombus in the distal segment of the left anterior descending artery.

A 23-year-old Chinese woman with a 3-year history of UC was admitted to the coronary care unit due to prolonged chest pain for 24 h. She had been treated with oral mesalazine for 2 weeks and methylprednisolone enemas (40 mg/d) for 4 days prior to admission. Her blood pressure and pulse were normal. Electrocardiogram showed sinus rhythm with pathological Q waves, ST segment elevation, and inverted T waves in leads V1–V5. Laboratory data revealed a cardiac troponin I of 3.3 ng/mL (relative index < 0.02 ng/mL), a CKMB of 111.0 ng/mL (relative index < 7.0 ng/mL), a myoglobin of 900.0 ng/mL (relative index < 112.0 ng/mL), and an N-terminal prohormone of brain natriuretic peptide of 4970 pg/mL (relative index < 450 pg/mL). Her echocardiographic examination revealed segmental ventricular wall motion abnormalities and an estimated left ventricular ejection fraction of 58%. In addition, a 1.7 × 0.6 cm moderate echogenic mass was found at the apex (see Fig. 1). The results of coronary angiography showed smooth angiographic appearance except for a well-formed thrombus in the distal segment of the left anterior descending artery with a 50% local stenosis and Thrombolysis in Myocardial Infarction grade III flow (see Fig. 2).

Her chest X-ray, abdominal, and pelvic ultrasound examinations were normal. Carotid and extremity vascular Doppler examinations revealed smooth appearance. Other abnormal laboratory data included a hemoglobin of 82.6 g/L (relative index 115–150 g/L), platelets of 457 × 10^−9^/L (relative index 125–350), a erythrocyte sedimentation rate of 59 mm/h (relative index < 15 mg/L), a C-reactive protein of 27 mg/L (relative index < 20 mg/L), a D-dimer of 547 μg/L (relative index < 300 mg/L), and a slightly decreased total protein. These abnormities could be explained on the basis of chronic UC. Other laboratory results including blood lipid and glucose, renal function, coagulation factors, and electrolytes were within normal range. Further tests revealed unremarkable female hormones, thyroid function, immunoglobulin, antiphospholipid antibodies, antinuclear antibodies, vasculitis antibodies, complement, antistreptolysin O, rheumatoid factor, and circulating lupus anticoagulant.

## 3 Discussion

Previous studies demonstrate that the main risk factors for young adults with MI include smoking, hyperlipidemia, family history of coronary artery disease, hypertension, diabetes mellitus, and obesity.^[1]^ Our patient reported no history of smoking, drinking, and no personal or family history of cardiovascular diseases or chronic inflammatory diseases. With these risk factors excluded, we speculate that UC and related medication may have played an important role in the development of MI in this young woman.

UC and Crohn disease are the 2 main entities of inflammatory bowel disease (IBD). Previous literatures have suggested possible causative links between IBD and coronary artery disease.^[2,3]^ A recent retrospective cross-sectional study showed that the incidence of thromboembolic events in IBD patients was increasing over the past decade and more arterial thrombotic events were observed compared with venous thrombotic events.^[4]^ Another population-based study also indicated that IBD patients suffer from an increased risk of cardiac arterial thromboembolic diseases.^[5]^ Similar results were found in a recent Danish nationwide cohort study showing that IBD is associated with increased risk of MI, stroke, and even cardiovascular death.^[6]^ This increased risk of coronary artery disease in IBD patients can be partly explained by hypercoagulable state and coronary atherosclerosis.^[7]^ With a smooth coronary artery appearance in our patient, we speculate that hypercoagulable state due to the chronic stage of UC might be responsible for the occurrence of coronary artery thrombus. Previous studies also demonstrated that, compared with male patients, coronary artery diseases are more common in female IBD patients,^[8]^ especially in those over the age of 40 years.^[9]^ But MI occurred in this female patient at an even younger age.

There were also some inconsistent results indicating that IBD is not associated with increased risk of MI compared with the general population.^[10,11]^ But these results should be interpreted with caution as they failed to control for other traditional risk factors of coronary artery disease.^[12]^

Corticosteroids may have some prothrombotic effects. It remains controversial whether the use of corticosteroids may add risk to coronary artery disease in IBD patients. A recent study suggested that corticosteroids use may reduce the odds of acute coronary syndrome in IBD patients.^[13]^ However, it has also been suggested that short-term and high-dose corticosteroids administration may lead to severe cardiovascular conditions,^[14]^ especially in elderly Crohn disease patients.^[15]^ Our patient received methylprednisolone enemas (40 mg/d) for 4 days before the occurrence of MI. We speculate that there might be a link between corticosteroids exposure and her coronary artery thrombus. But with few data available comparing enemas and oral administration, studies are still in need to further investigate these underlying mechanisms.

There were several previously reported cases describing MI in young patients with UC. Three of these cases, 2 men and 1 woman had MI due to nonatherosclerotic coronary thrombosis, which was quite similar to our patient.^[16–18]^ The difference was that all these 3 patients suffered an exacerbation of UC prior to the occurrence of MI, while our patient was in a chronic stage of UC. Another 2 cases, a young man and a young woman, developed MI in the chronic stage of UC. But the cause of MI was left ventricular thrombosis and there was no coronary atherosclerosis observed.^[19,20]^ Another young man was diagnosed of atherogenic MI after a recent flare of UC and enteropathic arthritis.^[12]^

Together with existing literature, the current case adds evidence supporting that coronary artery thrombosis in UC is a serious condition and can occur in the young population. UC and related medications may be responsible for the development of nonatherosclerotic MI. However, information in this regard is still scarce and further studies are needed to unravel the underlying pathophysiology.
